# GR-independent down-modulation on GM-CSF bone marrow-derived dendritic cells by the selective glucocorticoid receptor modulator Compound A

**DOI:** 10.1038/srep36646

**Published:** 2016-11-18

**Authors:** Andres E. Barcala Tabarrozzi, Luz Andreone, Julie Deckers, Carla N. Castro, María L. Gimeno, Laura Ariolfo, Paula M. Berguer, María Antunica-Noguerol, Ana C. Liberman, Sabine Vettorazzi, Jan P. Tuckermann, Karolien De Bosscher, Marcelo J. Perone

**Affiliations:** 1 Instituto de Investigación en Biomedicina de Buenos Aires (IBioBA) - CONICET - Partner Institute of the Max Planck Society, Buenos Aires, Argentina; 2 Receptor Research Laboratories, Nuclear Receptor Lab, VIB-Department of Medical Protein Research, VIB, Ghent University, Gent, Belgium; 3 Fundación Instituto Leloir, IIBBA, CONICET, Buenos Aires, Argentina; 4 Institute for Comparative Molecular Endocrinology, University of Ulm, Ulm, Germany

## Abstract

Dendritic cells (DC) initiate the adaptive immune response. Glucocorticoids (GCs) down-modulate the function of DC. Compound A (CpdA, (2-(4-acetoxyphenyl)-2-chloro-N-methyl-ethylammonium chloride) is a plant-derived GR-ligand with marked dissociative properties. We investigated the effects of CpdA on *in vitro* generated GM-CSF-conditioned bone marrow-derived DC (BMDC). CpdA-exposed BMDC exhibited low expression of cell-surface molecules and diminution of the release of proinflammatory cytokines upon LPS stimulation; processes associated with BMDC maturation and activation. CpdA-treated BMDC were inefficient at Ag capture via mannose receptor-mediated endocytosis and displayed reduced T-cell priming. CpdA prevented the LPS-induced rise in pErk1/2 and pP38, kinases involved in TLR4 signaling. CpdA fully inhibited LPS-induced pAkt_Ser473_, a marker associated with the generation of tolerogenic DC. We used pharmacological blockade and selective genetic loss-of-function tools and demonstrated GR-independent inhibitory effects of CpdA in BMDC. Mechanistically, CpdA-mediated inactivation of the NF-κB intracellular signaling pathway was associated with a short-circuiting of pErk1/2 and pP38 upstream signaling. Assessment of the *in vivo* function of CpdA-treated BMDC pulsed with the hapten trinitrobenzenesulfonic acid showed impaired cell-mediated contact hypersensitivity. Collectively, we provide evidence that CpdA is an effective BMDC modulator that might have a benefit for immune disorders, even when GR is not directly targeted.

Dendritic cells (DC) are professional antigen-presenting cells that constantly sense endogenous and exogenous danger signals in most tissues. DC play a key role linking the innate and adaptive immune responses due to their unique ability to induce activation of naïve and memory T-lymphocytes. Danger signals in peripheral tissues induce a differentiation program on DC, referred to as maturation. This process enables DC to migrate to lymph nodes, attract naïve T-lymphocytes and efficiently present antigens captured at the periphery. Furthermore, DC have strong influence on the T_helper (Th) profile adopted by activated T lymphocytes. Both the type and expression level of costimulatory molecules on the surface of DC at the site of the immunological synapse, as well as the production or absence of signature cytokines co-determine the induction of T-cell proliferation and drive Th cell differentiation^1^. It is widely known that endogenous stimuli or pharmacological treatments can alter DC, affecting the expression level of their surface membrane proteins and cytokine production, ultimately modifying DC functionality. Modulation of DC maturation and function is a fascinating process not only to understand the way in which these cells can drive immune responses but also to design immune therapeutic strategies for disorders involving immune responses, such as autoimmunity, cancer and transplantation. Understanding the biology of DC as well as their responses to *in vitro* manipulation is an intense field of research^2^.

Glucocorticoids (GCs) influence several biological processes including resistance to stress, regulation of intermediary metabolism, and immunosuppressive and anti-inflammatory effects^3^. GCs are potent endogenous immunomodulatory agents affecting differentiation and activation of lymphoid and myeloid cells^4^. In particular, GCs alter DC differentiation from progenitor cells and severely impair DC maturation and migration induced by inflammatory stimuli^5^,^6^. Synthetic GCs are widely used as anti-inflammatory and immunosuppressive drugs to treat several immune disorders including rheumatoid arthritis, asthma, eczema, and inflammatory bowel disease; they are also employed in organ transplantation. However, chronic administration of GCs induces severe side effects such as adrenal insufficiency, diabetes mellitus, osteoporosis, skin atrophy, growth failure, hypertension and glaucoma among others^7^. Immunomodulatory effects of GCs involve a GC-receptor (GR)-DNA independent interaction mechanism known as transrepression by which activated GR may act as a monomer interfering with inflammatory transcription factors such as NF-κB, AP1 and Stat5 by direct protein-protein interactions^8^,^9^. It is becoming increasingly clear that the full anti-inflammatory potential of GCs also depends on the action of GR as a genuine transcription factor, i.e. a transactivation mechanism in which activated GR dimerizes, binds to GC response elements (GRE) at the promoter or enhancer regions of GC-regulated genes and induces target genes^10^. However, as this mechanism also drives gluconeogenic gene expression, the diabetogenic side effect of GCs is also linked to transactivation^7^,^9^. These differential mechanisms have fueled the interest in the study and development of new GR-ligands with dissociative properties combining GCs’ anti-inflammatory properties with a reduced side effect profile.

Compound A (CpdA, (2-(4-acetoxyphenyl)-2-chloro-N-methyl-ethylammonium chloride) is a GR ligand without a steroidal structure unlike dexamethasone and prednisolone. It has been identified as a plant-derived dissociative GR-ligand that interferes with NF-κB by transrepression and unable to induce transactivation^11^. CpdA has been studied in several animal and cellular models showing potent anti-inflammatory properties, and reduced metabolic side effects^12^,^13^,^14^. The effects of CpdA on the immunomodulation of lymphocytes and macrophages have been documented *in vitro* and *in vivo*^13^,^15^,^16^,^17^.

Several lineage-specific transcription factors are required for naïve T-cell differentiation into distinct Th subpopulations. The master transcription factor TBX21 (T-box transcription factor, also known as T-bet) is overexpressed in T lymphocytes committed to the Th1 lineage. It enhances *IFNG* transcription, and at the same time represses *IL4* and *GATA-3*^18^,^19^. A CpdA-mediated inhibition of the Th1 phenotype was demonstrated to result from its suppression of the transcriptional activity of T-bet via a mechanism involving GR-mediated transrepression, similar to the effect described for classic GCs^9^.

In a model of experimental autoimmune neuritis, CpdA augmented Th2 cytokine and Foxp3 expression, concomitant with a down regulation of Th1 and Th17 cytokines. Furthermore, CpdA switched macrophages from the classically activated M1-type to the alternatively activated anti-inflammatory M2-type *in vitro*^13^. It is known that a GM-CSF-conditioned mouse bone marrow culture generates a heterogeneous population of CD11c^+^ MHCII^+^ dendritic cells and macrophages, which comprise BMDC^20^. In the current study, we generated BMDC *in vitro* employing a classic and widely used protocol containing GM-CSF as previously described^21^,^22^,^23^,^24^, and investigated the immunomodulatory effects of CpdA on the maturation and functionality of BMDC *in vitro* and *in vivo* upon an inflammatory challenge in comparison with the classical synthetic steroidal GR-agonist dexamethasone. *In vitro* generation of modulatory BMDC would be an invaluable tool in the therapy against allograft rejection and autoimmune diseases^20^.

## Results

### CpdA impairs the secretion of pro-inflammatory cytokines and up-regulation of costimulatory CD80 by LPS-stimulated GM-CSF- bone marrow-derived dendritic cells

**Figure 1:**
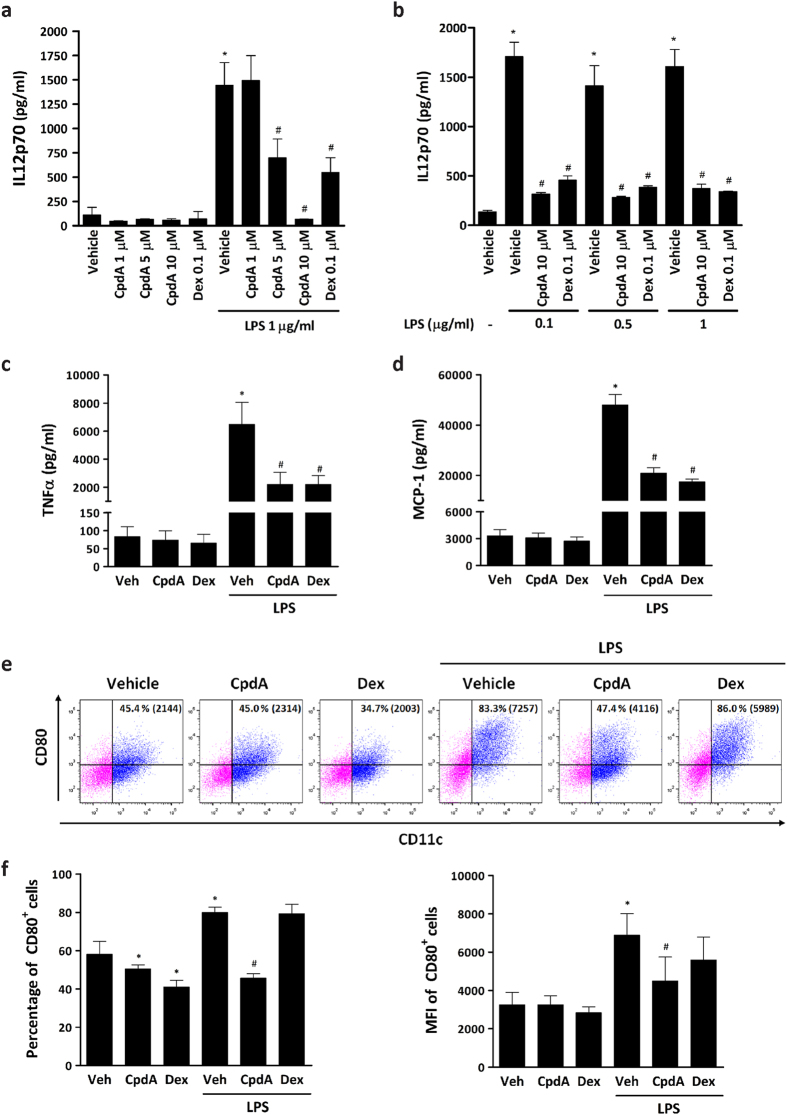
CpdA impairs LPS-induced pro-inflammatory cytokine secretion and up-regulation of CD80 by BMDC. BMDC were pretreated with vehicle, CpdA at indicated concentrations or Dex 0.1 μM for 1 h and stimulated with indicated amounts of LPS or left unstimulated. After 24 h, cytokine secretion was determined by ELISA and surface expression of CD80 was analyzed by flow cytometry. (**a**) Effect of different concentrations of CpdA (1–10 μM) on IL12p70 secretion by BMDC. (**b**) Analysis of 10 μM CpdA effect on IL12p70 secretion by BMDC challenged with different doses of LPS. (**c,d**) Effect of 10 μM CpdA on TNFα and MCP-1 secretion by BMDC. (**e**) Representative dot plots of BMDC after treatments. Upper right quadrants indicate the percentage of CD11c^+^ BMDC expressing CD80 and the mean fluorescence intensity (MFI) in brackets. (**f**) Statistical analysis of the percentage and MFI of CD80^+^ CD11c^+^ BMDC. Data are shown as mean ± SD of triplicate determinations from one representative out of two independent experiments (panels a–d) or as mean ± SD of three independent experiments (panel f). ^(*)^p < 0.05 vs. vehicle, ^(#)^p < 0.05 vs. vehicle + LPS.

We generated BMDC *in vitro* employing a widely used protocol containing GM-CSF + IL4 as previously described^21^,^22^,^23^,^24^. Based on a recent study describing that GM-CSF BMDC comprise a heterogeneous population that varies depending on culturing conditions, we first phenotypically characterized BMDC, harvested after 7 days in culture. In brief, we obtained two CD11c^+^ subsets (CD11c^+^ MHCII^low/int^CD11b^high^ and CD11c^+^ MHCII^high^CD11b^int^). Approximately 80% of the CD11c^+^ MHCII^high^CD11b^int^ subset resulted in CD115-CD135^+^ (GM-DC), which is in agreement with results reported by Helft *et al*.^20^ (Supplementary Figure S1). This heterogeneous BMDC model has been employed by several authors for a long time to successfully study the complex regulation of immunomodulatory processes^21^. A key feature of LPS-stimulated DC for the establishment of an efficient T-cell response against pathogens resides in their ability to produce pro-inflammatory cytokines^25^. It has been demonstrated that CpdA may be as effective as Dex exerting its anti-inflammatory effects on lymphocytes and macrophages. The effective concentration of CpdA reported *in vitro* varies between 1–10 μM depending on the cell line or patient cell material and the biologic readout assayed^11^,^26^,^27^. Therefore, we first investigated the optimal concentration of CpdA able to block the secretion of IL12p70 by LPS-stimulated BMDC *in vitro*. BMDC were pretreated with vehicle, CpdA at different concentrations or 0.1 μM Dex for 1 h followed by LPS challenge for 24 h, and supernatants analyzed by ELISA. The presence of CpdA in the culture medium did not affect the basal secretion of IL12p70 (Fig. 1a). As expected, LPS stimulation increased the secretion of IL12p70 by BMDC (>6-fold vs. vehicle). CpdA did not show any effect at the lowest concentration (1 μM CpdA). Pretreatment with 5 μM CpdA partially inhibited the IL12p70 secretion by LPS-stimulated BMDC. Finally, 10 μM CpdA blocked the secretion of IL12p70 by BMDC (Fig. 1a) independently of the LPS concentration used (Fig. 1b). Thus, 10 μM CpdA and 0.1 μM Dex as a control were adopted for the following experiments.

Culture supernatants of BMDC were quantified for TNFα and MCP-1 by ELISA (Fig. 1c,d, respectively). CpdA- and Dex-treated BMDC showed a reduction in TNFα and MCP-1 (up to 3-fold) secretion in response to the LPS-stimulus. Their basal secretion was not affected by either CpdA or Dex pre-treatment. IL10 was not detected in any experimental condition (data not shown).

In order to evaluate whether the continuous presence of CpdA before the LPS-challenge is necessary to achieve impaired secretion of pro-inflammatory cytokines, we assessed several treatment schemes. First, we pretreated BMDC for 1 h with either CpdA or Dex and then washed exhaustively before they were challenged with LPS during 24 h (Supplementary Figure S2). The transient presence of CpdA or Dex for only 1 h was enough to reduce LPS-stimulated IL12p70 secretion. Also, the action of CpdA 1 h before, simultaneously or 1 h after LPS-challenge effectively impaired IL12p70 secretion (Supplementary Figure S2). CpdA appeared however slightly more effective when administered 1 h before (519 +/− 8 vs. 2389 +/− 346 (veh) pg/ml of IL12p70) the LPS stimulus in comparison to the simultaneous treatment regimen (758 +/− 38 vs. 2389 +/− 346 (veh) pg/ml of IL12p70) or 1 h later treatment (1014 +/− 52 vs. 2389 +/− 346 (veh) pg/ml of IL12p70). Overall, CpdA treatment consistently reduces LPS-induced secretion of IL12p70 as a pivotal cytokine initiator of the Th1-biased T cell priming.

The process of DC maturation/activation enhances their immunogenicity by up-regulation of major histocompatibility complex (MHC)-peptide complexes and T lymphocyte costimulatory molecules (e.g., CD80) on the plasma membrane. Therefore, we investigated the effect of CpdA on the surface expression of CD80. BMDC were pretreated with vehicle, 10 μM CpdA or 0.1 μM Dex for 1 h followed by a challenge with 1 μg/ml LPS during 24 h. Surface expression of costimulatory CD80 molecules was analyzed by flow cytometry on CD11c^+^ (a commonly used DC surface marker) cells, excluding apoptotic cells by forward and side light scatter parameters, and 7-AAD staining. LPS stimulation increased the percentage of CD11c^+^ BMDC expressing CD80 as well as its mean fluorescence intensity (MFI, indicative of the expression of molecules per individual cell) (Fig. 1e,f). A reduction in the MFI values of CD80 was observed at low CpdA concentrations (1 μM CpdA, not shown) in comparison with LPS-challenged BMDC. However, a significant reduction in the percentage (80 +/− 3% of LPS-stimulated vs. 46 +/− 2% of CpdA-pretreated LPS-stimulated BMDC) and MFI (6883 +/− 1127 LPS-stimulated vs. 4500 +/− 1244 CpdA-pretreated LPS-stimulated BMDC) levels of LPS-activated CD11c^+^ BMDC expressing CD80 was evident at 10 μM CpdA (Fig. 1f).

### CpdA does not affect recovery of GM-CSF-bone marrow-derived dendritic cells

In addition to induce cell death of double positive thymocytes, supraphysiological levels of GCs inhibit thymopoiesis and accelerate apoptosis of mature T lymphocytes^28^,^29^. However, reports about GC-induced apoptosis in DC seem to show contradictory results depending on DC source, differentiation stage, the type and concentration of GCs used, and treatment duration^30^,^31^,^32^,^33^. In particular, it has been reported that DC treatment with Dex reduced viability and yield^34^. Therefore, we evaluated the recovery and viability of CD11c^+^ cells harvested on day 7 following GM-CSF plus IL4-treated bone marrow progenitors in culture. BMDC were pretreated with vehicle, 10 μM CpdA or 0.1 μM Dex for 1 h, and stimulated with 1 μg/ml LPS during 24 h. Recovery of CD11c^+^ BMDC was analyzed by flow cytometry. Viability was analyzed by Annexin-V staining followed by flow cytometry and trypan blue exclusion, in parallel. 60–80% of CD11c^+^ cells were harvested independently of the treatment received (Supplementary Figure S3). Importantly, CD11c^+^ BMDC recovery upon treatment with CpdA did not differ from the vehicle-control condition, independently of LPS stimulation. We found that treatment with CpdA resulted in a reduction in BMDC viability comparable to the one observed with Dex (58–70% of viable CpdA- or Dex-treated BMDC vs. 83–84% of viable vehicle-treated BMDC) (Supplementary Figure S3). The treatment with Dex followed by LPS challenge recovered BMDC viability, an effect that was not observed with CpdA (Supplementary Figure S3).

Using concentrations ≥ 1 μM Dex during 24 h in culture induced more than 60% of unviable BMDC (data not shown). All together, these results indicate that both CpdA and Dex, at the concentrations employed in this study, did not largely affect CD11c^+^ BMDC recovery and similarly diminished cell viability *in vitro*. The dramatic fall in pro-inflammatory cytokines secretion (e.g., ≥ 6-fold for IL12p70) and reduction of CD80 expression was not due to the loss of BMDC viability by CpdA since its transient presence for only 1h did not induce apoptosis of BMDC (data not shown) while showed a significant diminution of IL12p70 secretion comparable to the reduction without removal of CpdA (wash out experiment, Supplementary Figure S2).

### Effects of CpdA on the phenotypic maturation of BMDC

**Figure 2:**
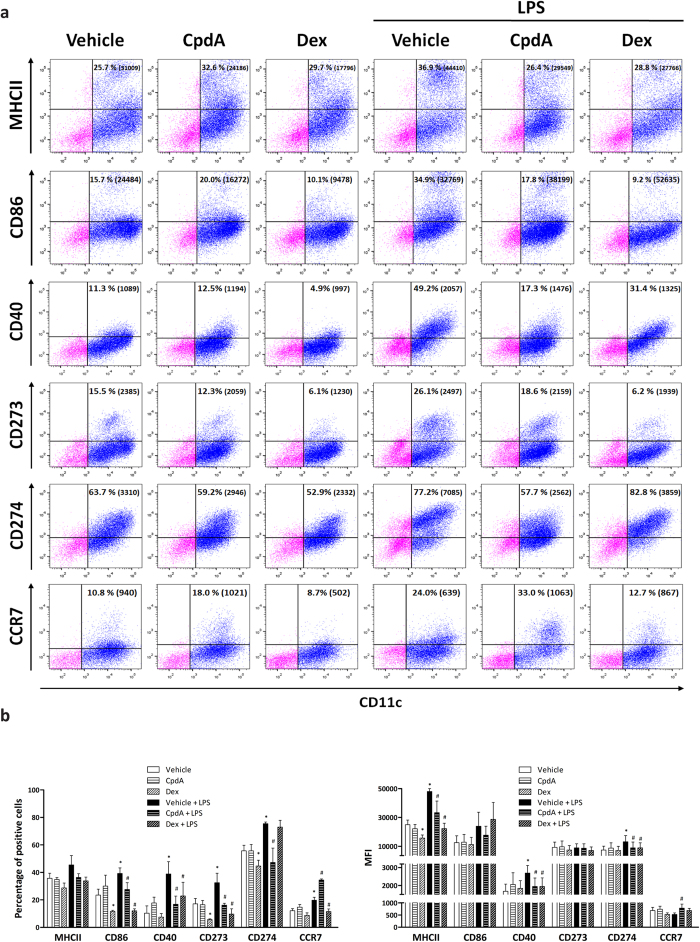
CpdA attenuates phenotypic maturation of BMDC. BMDC were pretreated with vehicle, CpdA 10 μM or Dex 0.1 μM for 1 h and stimulated with 1 μg/ml of LPS or left unstimulated during 24 h. (**a**) Surface phenotype of labeled BMDC with APC-CD11c mAb in combination with PE-labeled mAb against MHCII, CD86, CD40, CD273, CD274 or Alexa488-conjugated anti-CCR7 mAb followed by flow cytometric analysis. Upper right quadrants indicate the percentage of CD11c^+^ BMDC expressing CDs and the mean fluorescence intensity (MFI) in brackets. (**b**) Statistical analysis of the percentage and MFI of surface molecules in CD11c^+^ BMDC. Data are shown as mean ± SD of at least four independent experiments. ^(*)^p < 0.05 vs. vehicle, ^(#)^p < 0.05 vs. vehicle + LPS. Non-viable cells were excluded of the analysis by forward and side light scatter parameters, and 7-AAD staining.

The surface phenotype of CpdA-treated BMDC was analyzed by flow cytometry (Fig. 2a). LPS stimulation increased the percentage of CD40^+^, CD86^+^, CD273^+^, CD274^+^ and CCR7^+^ BMDC and simultaneously increased the expression level (MFI) of MHCII, CD40 and CD274 on the surface of BMDC (Fig. 2a,b). Both, CpdA and Dex diminished the LPS-induced increment in the percentage of CD40^+^, CD86^+^ and CD273^+^ BMDC, as well as MHCII, CD40 and CD274 MFI values. Comparing the effect of CpdA and Dex on immature BMDC, only the latter resulted in a decrease of MHCII^+^ (MFI), percentage of CD86^+^ and CD273^+^ CD11c^+^ expressing cells; effects that were not observed after CpdA treatment.

The chemokine receptor CCR7 was differentially modulated by CpdA and Dex after LPS-challenge. CpdA up-regulated, meanwhile Dex down-regulated the percentage of CCR7^+^ BMDC followed LPS-stimulation. This opposite effect on CCR7 expression exerted by both drugs might grant BMDC with a differential ability to migrate to the lymph nodes. No differences in CD45RB and CD54 expression were detected between all conditions analyzed (data not shown). With regard to classic GCs, these findings are in agreement in part with previous reports in that treatment with Dex induced a characteristic immature phenotype in DC^5^. In addition, our observations indicate that CpdA and Dex induced different phenotypic changes on immature and LPS-stimulated BMDC.

### Altered endocytic capacity of CpdA-treated BMDC

**Figure 3:**
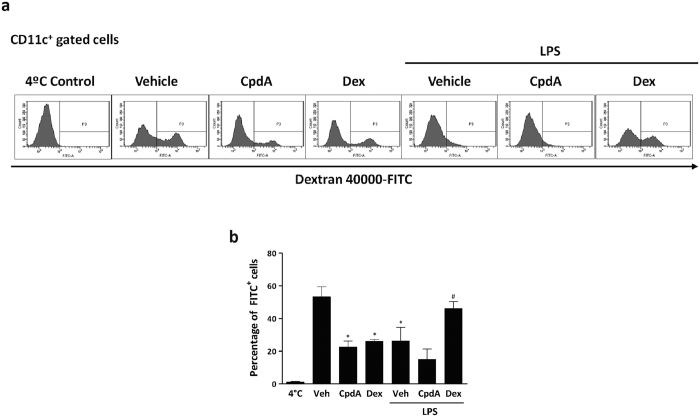
CpdA impairs the endocytic capacity of immature BMDC. BMDC were pretreated with vehicle, CpdA 10 μM or Dex 0.1 μM for 1 h, and stimulated or not with 1 μg/ml of LPS for 24 h. After extensive washing, BMDC were pulsed with Dextran-FITC during 1 h at 37 °C or 4 °C (control), washed and analyzed by flow cytometry. (**a**) Histograms show Dextran-FITC incorporation in CD11c^+^ gated cells from one representative out of six independent experiments. (**b**) Statistical analysis of the percentage of Dextran-FITC^+^ in CD11c^+^ gated cells. Data are shown as mean ± SD of six independent experiments. ^(*)^p < 0.05 vs. vehicle, ^(#)^p < 0.05 vs. vehicle + LPS.

Immature DC patrol peripheral tissues like sentinels sensing for the presence of pathogens and possess a high efficiency to internalize antigens through receptor-mediated endocytosis or phagocytosis. On the contrary, any stimulus that induces DC maturation might diminish their capacity of Ag uptake stabilizing peptide-MHC complexes at the plasma membrane for Ag presentation^35^. Therefore, we analyzed the capacity of BMDC for Ag uptake after CpdA treatment. BMDC were pretreated with CpdA or Dex for 1 h and stimulated or not with LPS for 24 h. Then, BMDC were harvested, extensively washed and pulsed with Dextran-FITC for 1 h (Fig. 3). The percentage of CD11c^+^ BMDC that incorporated Dextran-FITC was analyzed by flow cytometry, excluding Annexin-V^+^ cells. After 1 h of incubation, 53 +/− 6% of immature CD11c^+^ BMDC incorporated Dextran-FITC particles. The pretreatment with either CpdA or Dex significantly reduced the endocytic ability of immature BMDC, 23 +/− 4% or 26 +/− 1% (p < 0.05 vs. vehicle-treated), respectively (Fig. 3b). As expected, matured (LPS-stimulated) BMDC showed low endocytic activity (26 +/− 8% FITC^+^ CD11c^+^ BMDC). Dex-treatment before LPS stimulation preserved the endocytic capacity of BMDC that was substantially reduced in LPS-matured BMDC. On the other hand, CpdA pre-treatment did not prevent the LPS-induced reduction in Ag uptake. Taken together, these results indicate that both Dex and CpdA are able to reduce Ag uptake on immature BMDC but differently to Dex, CpdA was unable to restore endocytosis in LPS-activated/matured BMDC, indicating that CpdA impaired Ag uptake using a distinct unknown mechanism that the employed by Dex.

### CpdA reduces the T-cell stimulatory capacity of BMDC

**Figure 4:**
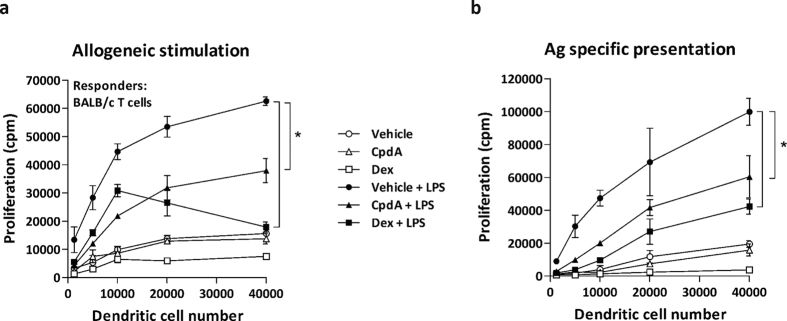
CpdA reduces the Ag presenting function of BMDC. C57BL/6 BMDC were pretreated with vehicle, CpdA 10 μM or Dex 0.1 μM for 1 h, and stimulated or not with 1 μg/ml of LPS for 24 h. (**a**) Allogeneic stimulation. Graded numbers of BMDC were co-cultured with 2 × 10^5^ responder BALB/c T cells during 3 days. (**b**) Graded numbers of BMDC pulsed with OVA_323-339_ peptide were used as stimulators and co-cultured with 2 × 10^5^ responder OT-II T cells during 3 days. T-cell proliferation was assayed by ^3^H-thymidine incorporation. Data are shown as mean ± SD of triplicate determinations from one representative out of two independent experiments. ^(*)^p < 0.05 vs. vehicle + LPS.

Due to the observed effects of CpdA on phenotype modulation, Ag uptake and reduction of pro-inflammatory cytokines secretion by BMDC, it was reasonable to speculate that CpdA might modulate their T-cell stimulatory capacity. First, in an allogeneic mixed lymphocyte reaction LPS-stimulated BMDC induced a strong T-cell proliferation, whereas the addition of either CpdA or Dex prior to LPS activation reduced their T-cell stimulatory response (Fig. 4a). When the effect of CpdA was monitored in an Ag-specific syngeneic assay to investigate the ability of BMDC to stimulate OVA_323-339_-peptide responder T-lymphocytes obtained from C57BL/6 OT-II TCR-transgenic mice, we also found a markedly reduced T-cell proliferation comparable to the effect exerted by Dex (Fig. 4b). As expected, immature BMDC in both syngeneic and allogeneic assays were poor inducers of T-cell proliferation. Thus, LPS-stimulated BMDC in the presence of CpdA result in antigen presenting cells with a poor capacity to induce T-cell proliferation. A similar effect was observed following Dex treatment of LPS-induced BMDC. IFNγ and IL4 secretion was analyzed from the supernatant of the allogeneic assays (data not shown). LPS-stimulated BMDC were potent inducers of IFNγ secretion (20237 +/− 120 pg/ml) and the combination with Dex exhibited a substantial reduction with an overall decrease in the IFNγ/IL-4 ratio (101 vs. 162 of LPS-BMDC). Contrary, CpdA-treated BMDC showed an increase in IFNγ/IL-4 ratio (241 vs. 162 of LPS-BMDC).

### CpdA impairs TLR4-stimulated signaling pathways in BMDC

**Figure 5:**
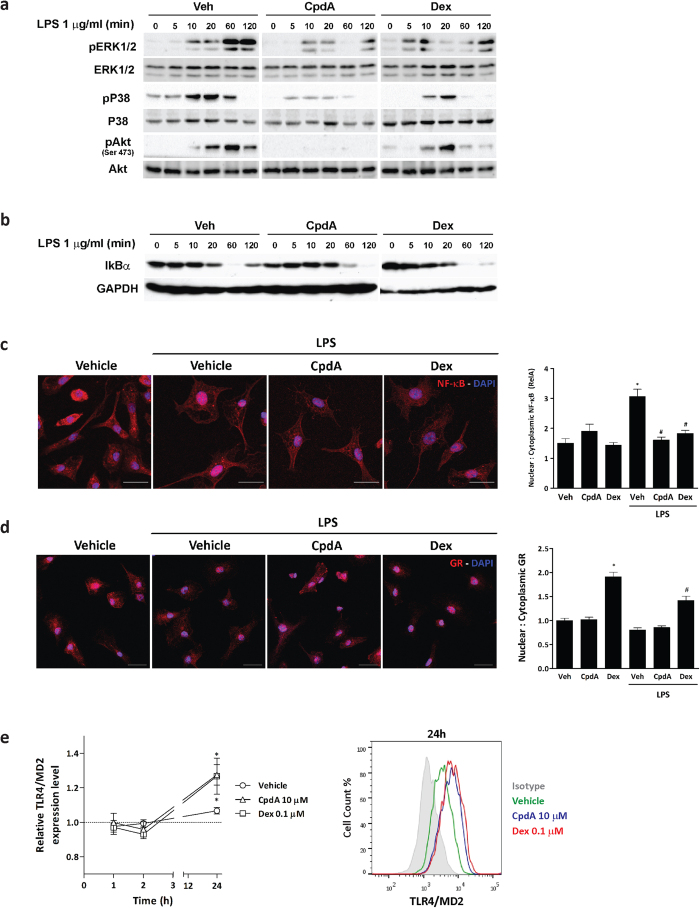
CpdA impairs TLR4 signaling pathways in BMDC. (**a**) BMDC were pretreated with vehicle, CpdA 10 μM or Dex 0.1 μM for 1 h and stimulated with 1 μg/ml of LPS or left unstimulated. After indicated time, levels of phosphoproteins were analyzed by Western blot. One representative out of three independent experiments is shown. (**b**) CpdA effect on IκBα expression. BMDC were treated as described in (**a**) and stimulated with 1 μg/ml of LPS. After indicated times, levels of IκBα were analyzed by Western blot. GAPDH was used as loading control. One representative out of three independent experiments is shown. (**c**) NF-κB nuclear translocation. BMDC were plated onto poly-L-lysine coated glass coverslips, pretreated with vehicle, CpdA 10 μM or Dex 0.1 μM for 1 h and stimulated with 1 μg/ml of LPS for 30 min or left unstimulated. NF-κB (RelA) expression was analyzed by immunofluorescence staining. Bar represents 20 μm. Quantification of nuclear:cytoplasmic ratio of NF-κB staining (red channel) is presented as mean ± SD from analysis of 5 separate high power field images. Results from one representative out of three independent experiments are shown. (*) p < 0.05 vs. vehicle, (^#^) p < 0.05 vs. vehicle + LPS. (**d**) GR nuclear translocation. BMDC were processed and treated as in (**c**) and GR expression was analyzed by immunofluorescence staining. Bar represents 20 μm. Quantification of nuclear:cytoplasmic ratio of GR staining (red channel) is presented as mean ± SD from analysis of 5 separate high power field images. Results from one representative out of three independent experiments are shown. ^(*)^p < 0.05 vs. vehicle, ^(#)^p < 0.05 vs. vehicle + LPS. **e)** Surface expression of TLR4/MD2 complex. BMDC were treated with vehicle, CpdA 10 μM or Dex 0.1 μM. After indicated times, BMDC were harvested and surface expression of TLR4/MD2 complex on CD11c^+^ cells was analyzed by flow cytometry. Relative TLR4/MD2 expression level was calculated as the MFI ratio for each condition at indicated times and for non-treated cells. Data are shown as mean ± SD of three independent experiments. ^(*)^p < 0.05 vs. vehicle. Representative histograms for each condition at 24 h treatment are shown, filled grey histogram represent isotype-matched control.

GCs interfere with TLR-induced kinases such as Erk1/2, JNK and Akt activated under specific conditions^36^. LPS binding to the TLR4/MD2 complex triggers activation of different kinases, including Akt and the MAPKs Erk1/2, JNK and P38, which may induce and/or fine-tune activation of down-stream inflammatory transcription factors such as NF-κB and AP1. Since CpdA interferes with downstream mediators following LPS activation on BMDC we asked whether it could affect TLR4 signaling in these cells. We analyzed by Western blot key phosphorylated kinases in LPS-activated BMDC that were pretreated with vehicle, 10 μM CpdA or 0.1 μM Dex. LPS increased the levels of pErk1/2, pP38 and pAkt in LPS-activated BMDC (Fig. 5a). Pretreatment with CpdA prevented the rise in pErk1/2, pP38 and pAkt, showing that CpdA impairs the activation of these kinases involved in TLR4 signaling. Dex diminished Akt activation after LPS treatment on BMDC. Unlike the effect of Dex, CpdA completely blocked phosphorylation of Akt at all-time points studied. Neither CpdA nor Dex affected the JNK pathway in BMDC (data not shown).

Inflammatory stimuli induce proteasomal IκBα degradation, releasing NF-κB. This transcription factor in turn translocates to the nucleus to activate inflammatory gene expression. After 20 min of BMDC stimulation with LPS, we observed a reduction of IκBα levels with its minimum expression at 60 min followed by a slow recovery starting at 120 min. CpdA delayed LPS-induced IκBα reduction reaching its minimum at 120 min post-stimulation (Fig. 5b). On the other hand, pretreatment with Dex increased initial IκBα expression but did not delay its LPS-induced degradation. Both CpdA and Dex inhibited with similar intensity NF-κB translocation to the nucleus at 30 min post-LPS stimulus (Fig. 5c). Then, we analyzed the subcellular localization of GR protein in BMDC. GR is located primarily in the cytoplasm of immature BMDC. Upon Dex treatment, GR was found to translocate from the cytoplasm to the nucleus independently of LPS stimulus. However, CpdA did not induce nuclear translocation of GR in BMDC (Fig. 5d).

To investigate whether the effect of CpdA on altering the expression of key intracellular components of LPS signaling in BMDC could be due to changes on TLR4 expression, BMDC were treated with vehicle, CpdA or Dex, followed by analysis of surface expression levels of TLR4/MD2 complex at different time points by flow cytometry (Fig. 5e). At the time when the LPS stimulus is supplied (1 h after CpdA- or Dex-treatment) we found the same levels of TLR4/MD2 at BMDC surface. Pretreatment with CpdA or Dex induced an increment of the TLR4 complex at 24 h in comparison to the vehicle-treated BMDC alone.

Ligand-induced down-regulation of the GR inhibiting glucocorticoid signaling has been postulated as a possible mechanism of GCs resistance^37^. Protein expression level of the main isoform of GR was not affected after 24 h of treatment with CpdA assessed by Western blot. Although Dex down regulated GR protein expression in BMDC, the presence of LPS preserves GR protein levels in the set-up that includes Dex (data not shown). Taken together, these results show that CpdA impairs TLR4 stimulation through a partial or complete inhibition of phosphokinases involved in inflammatory signaling, delays IkBα degradation, and inhibits nuclear translocation of NF-κB in DC, without inhibiting TLR4 and GR expression levels on BMDC. In contrast, synthetic Dex showed GR translocation to nucleus of stimulated BMDC.

### The immunomodulatory action of CpdA on BMDC is not affected by GR knockdown

**Figure 6:**
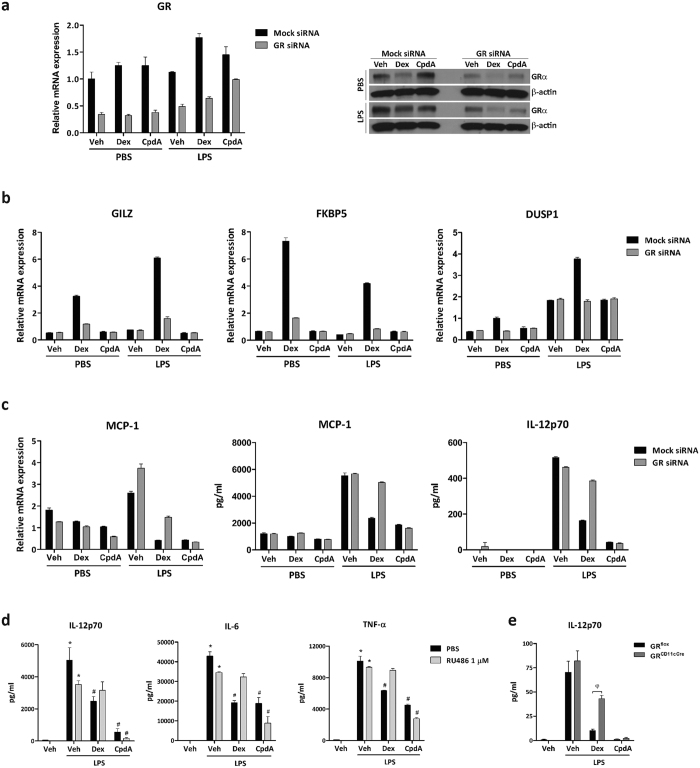
CpdA does not act exclusively through GR in mouse bone marrow-derived DC. For efficient RNA interference, 3 × 10^5^ BMDC were transfected with 150 nM mouse GR siRNA (Dharmacon) or 150 nM non-silencing control scrambled (mock) siRNA (RL, Dharmacon) by using the Amaxa^®^ Mouse Dendritic Cell Nucleofector^®^ Kit (Lonza). Sixteen hours after transfection, cells were pre-treated with vehicle, CpdA 10 μM or Dex 0.1 μM for 1 h before they were stimulated with 100 ng/mL LPS or PBS. After 24 h, (**a**) GR mRNA and protein expression were examined by real-time PCR (qRT-PCR) and Western blot, respectively. β-actin was used as loading control. One representative out of three independent experiments is shown. (**b**) RNA was isolated and levels of GILZ, FKBP5 and DUSP1 mRNA were measured by qRT-PCR. (**c**) RNA was isolated and levels of MCP-1 mRNA were measured by qRT-PCR, supernatant was collected and secretion of IL-12p70 and MCP-1 was quantified by ELISA. (**d**) BMDC were pretreated with vehicle, CpdA 10 μM or Dex 0.1 μM for 1 h with or without the GCs antagonist RU486 1 μM and stimulated with 1 μg/ml of LPS or left unstimulated. After 24 h, cytokines were determined with specific ELISAs. (**e**) BMDC obtained from GR^flox^ or GR^CD11cCre^ mice (four specimen of each genotype were run independently) were pretreated with vehicle, CpdA 10 μM or Dex 0.1 μM for 1 h and stimulated with 1 μg/ml of LPS or left unstimulated. After 24 h, IL-12p70 was determined with specific ELISA. Data is presented as mean ± SD of different determinations from one representative out of three independent experiments. ^(*)^p < 0.05 vs. vehicle, ^(#)^p < 0.05 vs. vehicle + LPS, ^(δ)^p < 0.05 GR^flox^ vs. GR^CD11cCre^.

It has been observed that CpdA exerts anti-inflammatory activities via activating GR in several cell types^11^,^12^,^17^,^38^. As we found that CpdA and Dex differentially modulate key mediators in the signaling pathways studied, we asked whether the GR is directly involved in the observed modulatory actions of CpdA on BMDC. To this purpose, we used siRNA knockdown of GR in BMDC. Figure 6a shows successful down-regulation of GR mRNA and GR protein levels when applying GR siRNA to BMDC (also, Supplementary Figure S4). mRNA level of GRE-driven genes, such as glucocorticoid-induced leucine zipper protein (GILZ, also known as TSC22 domain family protein 3), FKBP5 (FK506 binding protein 51) and DUSP1 (dual specificity phosphatase 1) were differently regulated by Dex and CpdA. Meanwhile Dex showed up-regulation of these mRNAs, CpdA had no effects on them (Fig. 6b, and Supplementary Figure S4).

Transrepression-dependent GR targets, here exemplified by LPS-activated MCP-1 and IL-12p70, were inhibited by both Dex and CpdA, an effect that was also apparent at the protein level (Fig. 6c and Supplementary Figure S4). Consequently, the partial loss of GR is expected to (at least partially) affect the suppression by both CpdA and Dex of these LPS-activated target genes. Surprisingly however, only the gene-repressive effect of Dex could be partially reverted following a knockdown of GR, suggesting GR-dependence only for the cytokine-inhibitory effect of Dex, but not of CpdA, in BMDC. In support of these findings, also GR blockade employing the GR antagonist RU486 1 μM was unable to inhibit suppression of inflammatory cytokines (TNFα, IL6, IL12p70) secretion by CpdA-pretreated LPS-stimulated BMDC (Fig. 6d). In contrast, this antagonist did reverse the inhibitory activity of Dex on LPS-stimulated BMDC.

Furthermore, to exclude that a remaining low expression of GR levels following siRNA silencing might be sufficient to still mediate GR-dependent effects, we analyzed BMDC generated from GR^CD11cCre^ mice in which the GR-encoding gene is completely ablated in CD11c^+^ DC^39^. We found that the secretion of IL-12p70 was inhibited by the action of CpdA in LPS-stimulated GR^CD11cCre^ as well as wild type BMDC whilst the inhibition on IL-12p70 secretion exerted by Dex on wild type BMDC after LPS-stimulation was reduced in GR^CD11cCre^ BMDC, implicating a GR-independent action of CpdA (Fig. 6e). All these findings reinforced the notion that immunomodulation of BMDC exerted by CpdA works mainly independent of GR.

### Reduced delayed-type hypersensitivity in mice adoptively transferred with hapten-sensitized CpdA-conditioned BMDC

**Figure 7:**
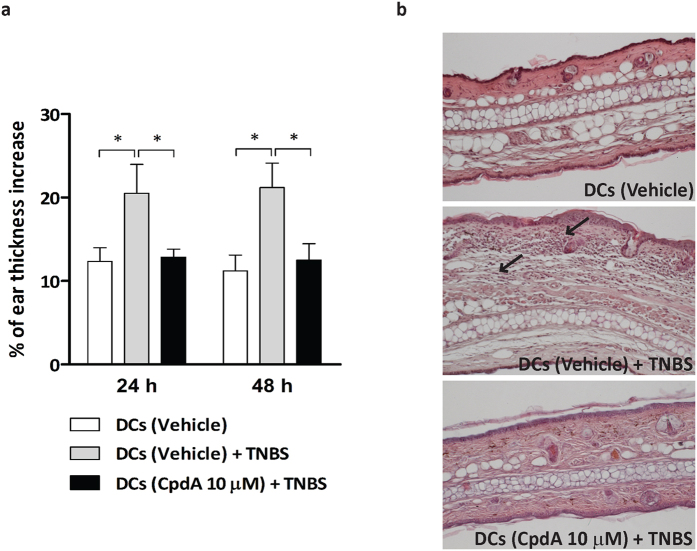
Reduced DTH response in mice sensitized by administration of CpdA-treated BMDC. BMDC were treated with vehicle or CpdA 10 μM for 24 h, washed and haptenized by incubation with TNBS 1 mM at 37 °C for 15 min. Haptenized-BMDC were subcutaneously injected (3 × 10^6^ cells/50 μl PBS/foot pad) into C57 mice; non-haptenized BMDC were used as control. After six days, mice were topically challenged with 20 μl of DNFB 0.4% w/v (olive oil:acetone, 4:1) in the right ears. Thickness of ears at 24 and 48 h were measured using a digital micrometer. (**a**) The percentage of ear thickness increase was calculated using the left ear as unchallenged control. Data are shown as mean ± SD of one representative (n = 5 mice per group) out of two independent experiments. ^(*)^indicates statistical differences with a p < 0.05. (**b**) Representative H&E staining of middle ear sections from each group of animals obtained 48 h after DNFB challenge (200X magnification). Arrows indicate inflammatory cell infiltrate in haptenized-BMDC group.

Having found that CpdA treatment promoted immature/semi-mature BMDC diminishing their capability to stimulate naïve T-cells *in vitro* we asked whether this observation may have *in vivo* relevance. We employed a DTH assay in which BMDC pre-incubated with TNBS (haptenized-BMDCs) were injected at the sensitization phase to evaluate their effects on naïve T-lymphocytes while testing whether the outcome was hapten-specific. Mice were sensitized by injection of haptenized-BMDC. As a specific control, one group of mice was sensitized with vehicle-treated BMDC (without the hapten). Six days later, the ear pinna was challenged topically with the hapten (DNFB) and the inflammatory response was evaluated at 24 and 48 h on the right (challenged) and left (control) ears by measuring the thickness of swelling. Mice sensitized with haptenized-BMDC showed a significant increase in swelling of the challenged ear detected at 24 and 48 h compared with those sensitized with non-haptenized-BMDC, indicating that the injected BMDC were able to mount a strong antigen-specific T-cell immune response (Fig. 7a). The ear thickness in mice sensitized with CpdA-conditioned haptenized-BMDC was significantly lower than in mice sensitized with vehicle-treated haptenized-BMDC (p < 0.05). The ears were subjected to microscopic analysis 48 h after DNFB challenge. Consistent with the increased ear swelling, histological examination of the ears in animals sensitized with vehicle-BMDC and challenged with hapten showed dermal edema, epidermal hyperplasia, leukocytes infiltration and vasodilation (Fig. 7b). On the other hand, animals that were sensitized with CpdA-BMDC and challenged with the DNFB showed mild ear edema similar to the control group challenged with vehicle alone (acetone/olive oil). These results indicate that CpdA-treated BMDC possess a reduced antigen-specific T-cell priming capability *in vivo*.

## Discussion

In this work we describe for the first time the ability of CpdA, identified before as a selective GR-ligand, to modulate GM-CSF bone marrow-derived DC (BMDC) phenotype and functionality. We found that particular BMDC-modulatory effects of CpdA might be explained by a GR-independent (nongenomic) inactivation of the NF-κB intracellular signaling pathway following TLR4 activation, associated with pErk1/2, pP38, and pAkt upstream signaling.

Because no marker distinguishes DC and macrophages unequivocally, the existence of a homogeneous population after GM-CSF activated bone marrow-derived progenitor cells has been questioned^20^. With this concept in mind, here we described the effects of CpdA based on a BMDC heterogeneous CD11c^+^ population that has been demonstrated to be useful for immunomodulatory studies since a long time^21^,^22^,^23^.

In addition to the effects of GCs on activated T cells, it is known that GCs exert immunomodulatory activity on DC^5^,^25^. Besides their anti-inflammatory action, GCs might also exert several adverse effects, with hyperglycemia and hyperinsulinemia being one of the major drawbacks of their long-term use mainly through a direct transcriptional effect of activated GR on gluconeogenic enzymes by transactivation mechanisms. Therefore, the development of safer GR targeting compounds with the dissociated action of a selective GR agonist, the so-called dissociated ligands, is of major research interest for translational medicine.

Tolerogenic DC are associated with low expression levels of costimulatory molecules (e.g., CD80, CD86). In the generation of DC for cell-based therapy the need for an impaired CD40 signal is recognized^40^. We demonstrate that CpdA impairs LPS-induced maturation of BMDC by diminishing costimulatory molecules. The effects of CpdA on BMDC differ in several aspects with those treated with classical GCs, such as Dex^25^. We observed a down-regulation of GR protein levels after Dex treatment of BMDC, an effect that was not observed after CpdA treatment. It is documented that down-regulation of GR protein in GCs-treated cells may occur by multiple mechanisms and it is cell-type specific. The primary one appears to be a decreased GR protein half-life after hormone binding to the GR^41^.

The treatment of BMDC with CpdA causes a remarkable reduction of the secretion on several pro-inflammatory cytokines. In fact, BMDC secrete a wide array of immunological relevant soluble agents and, it is well established that the cytokine profile expressed depends on the microenvironment in which they are activated^42^. We found that CpdA-treatment reduces LPS-triggered secretion on IL-12p70 by BMDC. The cytokine IL-12p70 is responsible for the development of Th1 lymphocyte differentiation and proliferation^43^. In line with these observations, CpdA-treated BMDC show poor T-cell stimulatory capacities using an allogeneic mixed lymphocyte reaction and Ag-specific syngeneic assays *in vitro*. Upon using a DTH assay to evaluate *in vivo* Th1 cell-mediated responses producing high levels of IFNγ, our *in vivo* results showed consistency with the finding that CpdA-treated BMDC induce poor activation of naïve T cells *in vitro*. The fact that CpdA increases the expression of the chemokine receptor CCR7 on BMDC *in vitro* might suggest that the migratory activity of BMDC to lymph nodes is unaffected, in contrast to the action of GCs on these cells. Low levels of CCR7 after Dex treatment disable DC to migrate towards a CCL19 gradient, one of its specific ligands of CCR7^44^. Future studies are needed to further evaluate the effect of CpdA on BMDC migratory activity.

We have reported that the mechanism of action of CpdA on T-lymphocytes arises from a selective GR modulation. Inhibition of *IFNG* gene expression and secretion of IFNγ following selective CpdA-mediated GR modulation involves the inhibition of T-bet activity in T lymphocytes^15^. Although IFNγ is considered a signature cytokine of CD4^+^ Th1, CD8^+^ and NK cells it has been demonstrated that antigen-presenting cells synthesize this pro-inflammatory cytokine as well, in particular DC. T-bet expression by DC is rapidly stimulated by IFNγ, and secretion of IFNγ by DC is also positively influenced by IL-12p70 stimulation^45^. IL-12p70 is pivotal for Th cell differentiation into the Th1 phenotype, and the synthesis and secretion of IFNγ by Th1 lymphocytes. Here, we found a strong inhibition of IL-12p70 and TNFα secretion by LPS-stimulated CpdA-conditioned BMDC and also, the chemoattractant MCP-1. The establishment of a positive feedback loop in the communication between DC and T cells is important to maximize type 1 immunity. Therefore, it is tempting to speculate that CpdA might exert the same modulatory effects on DC *in vivo*, explaining in part their therapeutic action on T-cell mediated diseases such as collagen-induced arthritis^12^.

DC express different pattern recognition receptors (PRRs) that recognize pathogen-associated molecular patterns (PAMPs), like LPS^46^. Antigen recognition involves receptor-mediated endocytosis or phagocytosis that subsequently results in DC maturation and migration to secondary lymph nodes for T cell priming^47^. We found that CpdA impairs Ag uptake to a similar extent as Dex on immature BMDC. However, in contrast of the regained endocytic funtion of LPS-BMDC exerted by Dex, CpdA-treated LPS-BMDC remained with very low endocytic ability. Besides several differential effects observed by the action of both drugs on BMDC phenotype and function, the strongest effect of CpdA may be related to the specific genes that are targeted when it function as a transcription factor repressor that may include genes that affect the capacity of BMDC to take up antigens. In view of the fact that CpdA-BMDC were poor T cell stimulators we might explain this observation in part to their low antigen uptake capacity. The association with lowered expression levels of molecules involved in T cell stimulation such as MHCII, CD40, CD80 and CD86 may also account for low endocytic capacity after CpdA exposure. Several agents have been reported as modulators of DC function affecting the expression of costimulatory molecules giving them an immature phenotype such as IL10, aspirin and 1,25-dihydroxyvitamin D3^48^,^49^,^50^. However, for most of them, the induction of an immature phenotype as well as the poor stimulatory T cell response was associated with enhanced endocytic antigen ability.

LPS acts through the innate receptor toll-like receptor 4 (TLR4) activating the MyD88 and TRIF pathways, inducing phosphorylation of several intracellular kinases including the IκBα kinase, the extracellular signal-regulated kinases Erk1/2, P38 MAPK, c-Jun N-terminal kinases and phosphatidylinositol-3-kinase (PI3K)-protein kinase B (Akt)^51^. Immune cell activation is linked to changes in cellular metabolism. Krawczyk *et al*. demonstrated that TLR signaling exerts a metabolic reprogramming allowing full maturation and functionality of DC^52^. In fact, TLR signaling favors glycolytic metabolism over mitochondrial oxidative phosphorylation by activating the PI3K-Akt pathway that allows a rapid provision of ATP to maintain cell viability and anabolic demand during an immune response. In addition to PI3K, Akt is essential for DC survival and function after inflammatory stimuli^53^. Mammalian/mechanistic target of rapamycin (mTOR) activated by the PI3K/Akt signaling pathway, controls glycolysis and metabolism, and regulates cell activation and proliferation. Our data indicate that CpdA treatment of BMDC strongly inhibits LPS-induced pAkt_Ser473_ which is an upstream activator of mTOR, suggesting that CpdA might induce a metabolic switch to mitochondrial oxidative phosphorylation and acceleration of catabolism that has been associated with the generation of tolerogenic DC^54^. Dex actually reduced LPS-induced pAkt in BMDC far less than CpdA. In this respect, pharmacologic treatments to induce tolerogenic DC such as the use of Dex or Vitamin-D3 have been described that also affect DC metabolism^55^. Several studies have reported that MAPK signaling pathways modulate DC function and survival^53^,^56^. We therefore investigated whether CpdA interferes with LPS-induced activation of these pathways in BMDC. The increased expression of pErk1/2 and pP38 after LPS stimulation was markedly inhibited by both CpdA and Dex treatment on BMDC. However, phosphorylated JNK was apparently not affected by either CpdA or Dex on BMDC (data not shown). NF-κB signaling is involved in LPS-induced DC maturation and inhibition of this pathway suppresses DC maturation and function^57^. The levels of IκBα, the predominant inhibitory molecule of NF-κB, were found to be augmented in the CpdA-BMDC changelled with LPS. CpdA and Dex presented different kinetics in the degradation of IκBα in BMDC, probably as a result of differences in their mechanisms of action. Dex may induce IκBα transactivation through GR binding to a GRE within its promoter region^58^. This probably explains the high IκBα levels after pretreatment with Dex for 1 h. Unlike Dex, CpdA does not induce transactivation. However, CpdA treatment does lead to a delay in IκBα degradation, possibly by non-genomic effects of monomeric GR activation. CpdA has initially been described as a fully dissociated GR ligand. However, using GR knockdown analysis combined with pharmacological GR blockade and the use of GR^CD11cCre^ mice to obtain BMDC lacking GR, we show in this study that the immunomodulatory activity of CpdA on BMDC was able to persist in the absence of fully functional GR levels. Moreover and opposite to DEX, CpdA inhibited nuclear translocation of GR in BMDC. Particular GR-independent effects of CpdA have been reported before and are hence not restricted to DC. In a recent report studying airway smooth muscle cells, it was observed that CpdA inhibits the secretion of GC-resistant chemokines in a GR-independent manner^59^, again emphasizing benefit in absence of GR as a target of CpdA.

It has been shown that CpdA functions as not only a GR modulator but also an androgen receptor (AR) antagonist^60^. Bone marrow-derived DC express AR and progesterone receptors (PR)^61^ and their activation act as suppressor of DC function. Therefore, we cannot discard that a possible mechanism of action of CpdA might in part be associated to progesterone/androgen ligation to their respective receptors on BMDC. Inflammatory signaling pathways affected by CpdA can also include a GR-independent component. Indeed, the effects of CpdA on TNFα-induced MAPK activation occurred independently of GR in rheumatoid arthritis synovial fibroblasts^62^, albeit here CpdA-mediated cytokine suppression, i.e. of IL1β, did rely on GR. This report may encourage further research into the *in vivo* function of CpdA-treated BMDC in relation to their potential immunomodulatory ability on undesired allogeneic or autoimmune responses, in which regulatory DC have shown tolerogenic properties. By using CpdA, or preferably more stable analogues hereof, a suppression of DC function may be achieved in order to efficiently control specific undesired immune responses.

## Methods

### Animals and reagents

Six- to 8-week-old C57BL/6J and BALB/c mice were purchased from Bioterio Central, FCEyN, University of Buenos Aires. The B6.Cg-Tg(TcraTcrb)425Cbn/j (OTII) mice were obtained from Fundación Instituto Leloir. The GR^CD11cCre^ (Nr3c1^tm2Gsc^Tg(Itgax-cre)1-1Reiz) and GR^flox^ (Nr3c1^tm2Gsc^) mice were obtained as previously described^39^,^63^. Studies were approved by the Institutional Care and Use Committee (CICUAL #0001) FCEyN, University of Buenos Aires. Animal care and experimental procedures were carried out in accordance with the guidelines of the Institutional Care and Use Committee of FCEyN-Univ. of Buenos Aires. CpdA was synthesized as described^26^. Dexamethasone (Dex), RU486, LPS, 2,4 dinitro-1-fluorobenzene (DNFB) and 2,4,6-trinitrobenzenesulfonic acid (TNBS) were purchased from Sigma-Aldrich.

### *In vitro* generation of GM-CSF bone marrow-derived dendritic cells (BMDC)

BMDC were generated *in vitro* from mouse bone marrow precursors cultured with GM-CSF and IL4 as described previously^21^. At day 7, cells were subjected to CpdA or Dex and LPS treatments and subsequent analysis.

### Cytokines quantification

Detection of IFNγ, IL4, IL6, IL10, IL12p70 and TNFα in BMDC supernatants was performed according to manufacturer´s protocol using Biolegend ELISA sets. MCP-1 secretion was determined using Ready-SET-Go! ELISA Kit (eBioscience).

### GR silencing by Nucleofection of BMDC with target-specific siRNA

C57Bl/6 bone marrow derived BMDC were harvested on day 8. For efficient RNA interference, cells were transfected with mouse GR siRNA (Dharmacon) or non-silencing control scrambled (mock) siRNA (RL, Dharmacon) by using the Amaxa^®^ Mouse Dendritic Cell Nucleofector^®^ Kit (Lonza). Sixteen hours after RNA interference, cells were pre-treated with DEX or CpdA or solvent control and stimulated with LPS or PBS as a control.

### qRT-PCR

RNA was isolated from BMDC by using RNeasy Micro Kit (Qiagen) and mRNA was reverse transcribed to cDNA with the PrimeScript RT kit (TaKaRa).

### Phenotypic dendritic cells analysis and endocytosis assay

Inspecific binding on BMDC were blocked with normal bovine serum and incubated with the following PE-conjugated mAbs: anti-IA^d^, -CD80, -CD86, -CD40, -CD54, -CD45RB, -CD273, Alexa488-conjugated anti-CD197(CCR7) and biotin anti-mCD11c followed by APC-streptavidin (Biolegend). As a negative control isotype-matched irrelevant mAbs were used. Apoptosis was assessed by phosphatidylserine exposure analysis using PE-Annexin V staining (BD Biosciences) according to manufacturer’s instructions. Analysis was performed using FACS DIVA6. For endocytosis assay, BMDC (1 × 10^6^ cells) were incubated with 10 μg/ml FITC-Dextran (MW 40000, Life Technology, Molecular Probes) at either 37 °C or 4 °C for 1 h. Endocytosis was stopped by extensive wash in ice-cold 0.1% sodium azide-1% FCS-PBS and cells were stained for surface CD11c^+^, as described above.

### SDS-PAGE and Western blot analysis

BMDC were pretreated with vehicle, CpdA 10 μM or Dex 0.1 μM for 1 h and stimulated with 1 μg/ml of LPS for 0, 5, 10, 20, 60 and 120 min. Cells were harvested on ice-cold PBS, washed and lysed in 50 mM sodium phosphate/1% v/v SDS/40 mM 2-ME/2 mM EDTA buffer and conserved at −20 °C until use. Proteins were subjected to SDS-PAGE and blotted with antibodies against pErk1/2, Erk1/2, pJNK, JNK, pP38, P38, pAkt, Akt (Cell signaling), IkBα, GAPDH and GR (Santa Cruz) followed by the appropriate HRP-conjugated secondary antibodies (Bio-Rad). Staining was developed by ECL (Pierce Biotechnology).

### Confocal microscopy

BMDC were harvested, cultured onto poly-L-lysine coated glass coverslips and fixed by cold methanol. Cells were incubated with the following primary antibodies: anti-NFκB p65 (RelA) or anti-GR (M-20) (Santa Cruz). Secondary antibodies used at 1/200 dilution were either anti-goat or anti-rabbit Alexa Fluor 647 conjugated dye (Life Technology). The coverslips were mounted on slides with Mowiol. Images were acquired on a Zeiss Axio Observer Z1 LSM 710 Confocal Microscope (Carl Zeiss Microscopy GmbH). Data acquisition was performed with ZEN Black 2011 software and quantification using Fiji software.

### Contact hypersensitivity

BMDC were treated with vehicle or CpdA 10 μM for 24 h, haptenized with 1 mM TNBS and subcutaneously injected (3 × 10^6^ cells/50 μl of PBS/footpad) into C57Bl/6 mice. Non-haptenized vehicle-treated BMDC were used as a specificity control. After 6 days, mice were challenged with 20 μl of DNFB (0.2% in acetone/olive oil, 1:4; Sigma-Aldrich) on the right ear pinna. Thickness of the right (challenged) and left (control) ear pinna was measured with a digital micrometer at 24 h and 48 h after challenge. Swelling quantification was determined as the difference in the thickness between the right and the left ear pinna and expressed as percentage increase of ear thickness (mean, SE). Ear samples (5–6 mm) were fixed in 4% formaldehyde, embedded in paraffin, and processed for staining with H&E. Images were acquired on a Zeizz Axio Vert. A1 Inverted Microscope (Carl Zeiss Microscopy GmbH). Each group consisted of five mice and the experiment was performed twice.

### Antigen-specific presentation assay

C57Bl/6 BMDC (stimulator cells) were co-cultured with OTII nylon-wool purified naïve T lymphocytes (responder cells). BMDC (10^6^ cells/ml) were treated with either Dex or CpdA and LPS as mentioned before for 24 h, washed, pulsed with 1 μg/ml of OVA_323-339_ peptide for 2^1/2^ h and co-cultured at different ratios with 2 × 10^5^ responder T cells in 200 μl/well round-bottom 96-well plates in complete RPMI medium for three days. The last 18 h wells were pulsed with 1 μCi of ^3^H-thymidine. Cells were harvested on filter mats of glass fiber paper (Skarton Instruments) using a semiautomatic cell harvester. Glass fiber filters were air-dried and each dot was embedded in 1 ml of Optiphase scintillation liquid. The radioisotope incorporation was determined using a beta scintillation counter.

### Mixed lymphocyte reaction

BALB/c splenic T lymphocytes were enriched by passage through nylon wool columns and then used as responders (2 × 10^5^ cells/well) in round-bottom 96-well plates with graded numbers of C57Bl/6 BMDC and cultured for 72 h in complete RPMI. Proliferation of T cells was determined as above.

### Statistical analysis

Results are presented as mean±SD. Comparison between groups were carried out using paired or unpaired Student´s t-test or ANOVA followed by Bonferroni´s multiple comparison test, as appropriate. A p < 0.05 was considered to indicate a statistically significant difference. All statistical analyses were performed using GraphPad Prism version 5.0 Software.

## Additional Information

**How to cite this article**: Barcala Tabarrozzi, A.E. *et al*. GR-independent down-modulation on GM-CSF bone marrow-derived dendritic cells by the selective glucocorticoid receptor modulator Compound A. *Sci. Rep.* **6**, 36646; doi: 10.1038/srep36646 (2016).

**Publisher’s note:** Springer Nature remains neutral with regard to jurisdictional claims in published maps and institutional affiliations.

## Supplementary Material

Supplementary Information
