# Supplementary material for: GR-independent down-modulation on GM-CSF bone marrow-derived dendritic cells by the selective glucocorticoid receptor modulator Compound A
Source: Sci Rep. 2016 Nov 18;6:36646. doi: 10.1038/srep36646 (PMC5114550; doi:10.1038/srep36646)
Supplement: Supplementary Information [file srep36646-s1.pdf]

GR-independent down-modulation on GM-CSF bone marrow-derived dendritic cells by the selective glucocorticoid receptor modulator Compound A

Andres E. Barcala Tabarrozzi[1§], Luz Andreone[1§], Julie Deckers[2], Carla N. Castro[1], María L. Gimeno[1], Laura Ariolfo[1], Paula M. Berguer[3], María Antunica Noguerol[1], Ana C. Liberman[1], Sabine Vettorazzi[4], Jan P. Tuckermann[4,] Karolien De Bosscher[2] and Marcelo J. Perone[1*]

[§] *These authors contributed equally to this work.*

[1] *Instituto de Investigación en Biomedicina de Buenos Aires (IBioBA)-CONICET-Partner Institute of the Max Planck Society, Buenos Aires, Argentina.*

[2] *Receptor Research Laboratories, Nuclear Receptor Lab (NRL), VIB Department of Medical Protein Research, Ghent University, Ghent, Belgium.*

[3] *Fundación Instituto Leloir, IIBBA, CONICET, Buenos Aires, Argentina.*

[4] *Institute for Comparative Molecular Endocrinology, University of Ulm, Ulm, Germany.*

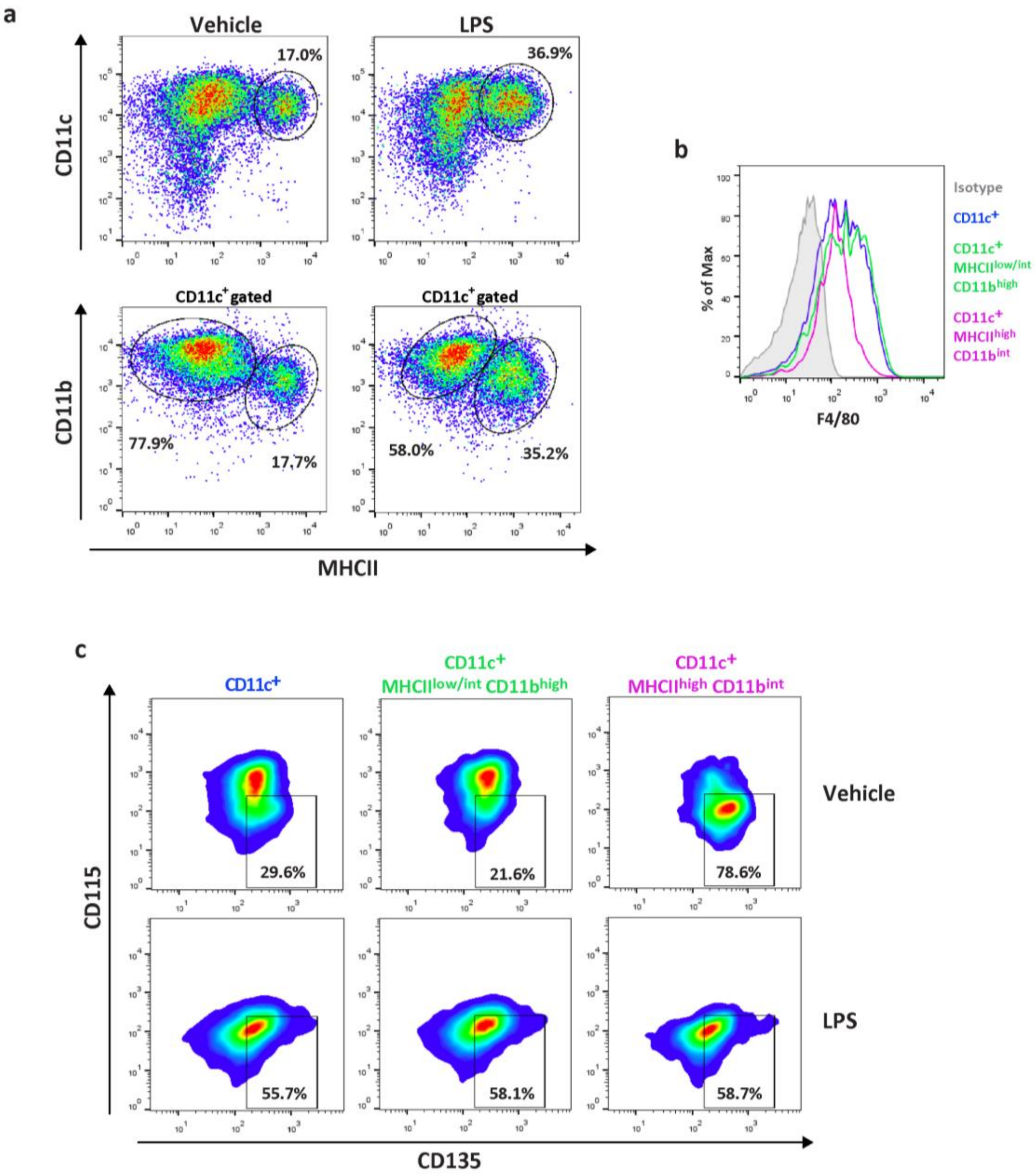

Supplementary Figure S1: Phenotype characterization of GM-CSF-conditioned bone marrow derived dendritic cells (BMDC). Representative flow cytometry analysis of GM-CSF+IL4-stimulated BM cultures harvested after 7 days in culture. **a)** CD11c$^+$ cells were analyzed on the basis of CD11b and MHCII expression under basal and LPS-stimulated conditions. Circles enfold cell sub-populations and numbers indicate the percentage of cells in each population. **b)** Histograms showing the expression of F4/80 by CD11c$^+$ (blue line), CD11c$^+$MHCII$^{low/int}$CD11b^{high} (green line) and CD11c$^+$MHCII^{high}CD11b^{int} (purple line) subpopulations. Filled histogram depicts isotype-irrelevant specificity control. **c)** CD115 and CD135 expression on CD11c$^+$, CD11c$^+$MHCII$^{low/int}$CD11b^{high} and CD11c$^+$MHCII^{high}CD11b^{int} BMDC subpopulations. Boxes represent CD115$^-$CD135$^+$ subpopulations with corresponding percentage of cells.

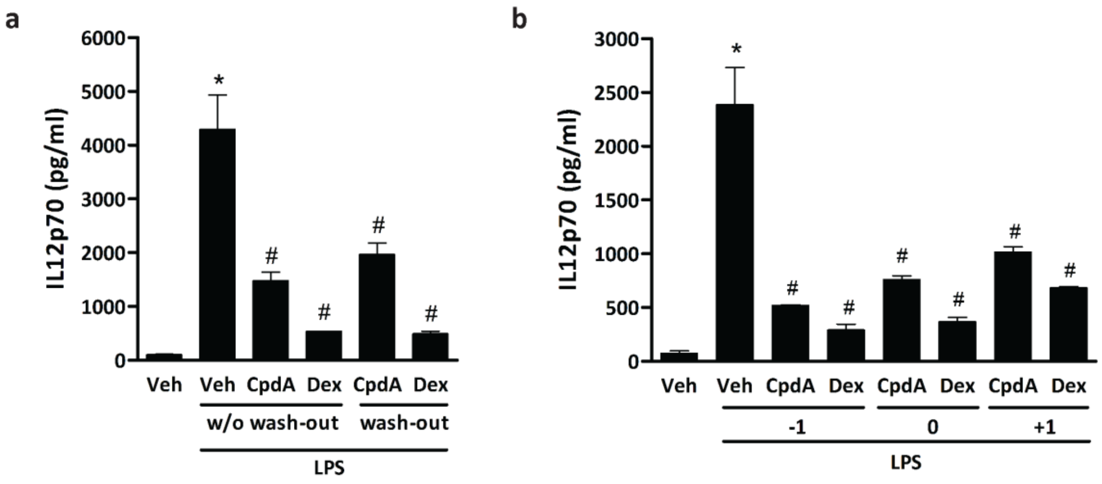

Supplementary Figure S2: CpdA impairs IL12p70 secretion by BMDC. Evaluation of different treatment schemes. a) The transient presence of CpdA impairs IL12p70 by LPS-challenged BMDC. Cells were pretreated with vehicle, CpdA 10µM or Dex 0.1µM for 1h and then, washed exhaustively before they were challenged with LPS. After 24h, secreted IL12p70 was determined by ELISA. w/o wash-out (without wash-out). **b)** CpdA impairs IL12p70 secretion by BMDC when it was applied 1h before, simultaneously or 1h after LPS 1µg/ml. After 24h, secretion levels of IL12p70 were determined by ELISA. Data are shown as mean ± SD of triplicate determinations from one representative out of two independent experiments. (*) $p < 0.05$ vs. vehicle, (#) $p < 0.05$ vs. vehicle + LPS.

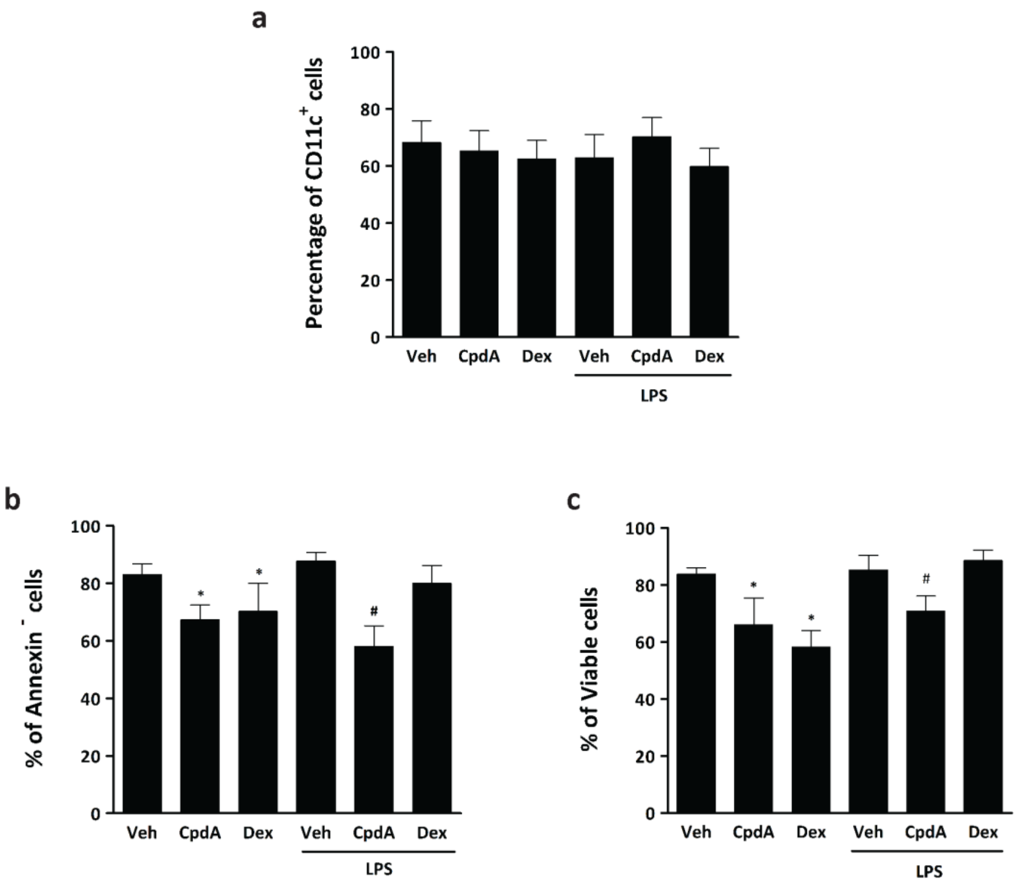

Supplementary Figure S3: Effect of CpdA on the recovery and viability of BMDC in culture. BMDC were pretreated with vehicle, CpdA 10µM or Dex 0.1µM for 1h and stimulated with 1µg/ml of LPS or left unstimulated. After 24h, **a)** the percentage of CD11c^{+} cells was analyzed by flow cytometry, **b)** the percentage of Annexin-V^{-} BMDC was analyzed by flow cytometry, **c)** the percentage of viable cells was determined by Trypan Blue exclusion. Data are shown as mean ± SD of at least four independent experiments. (*) $p < 0.05$ vs. vehicle, (#) $p < 0.05$ vs. vehicle + LPS.

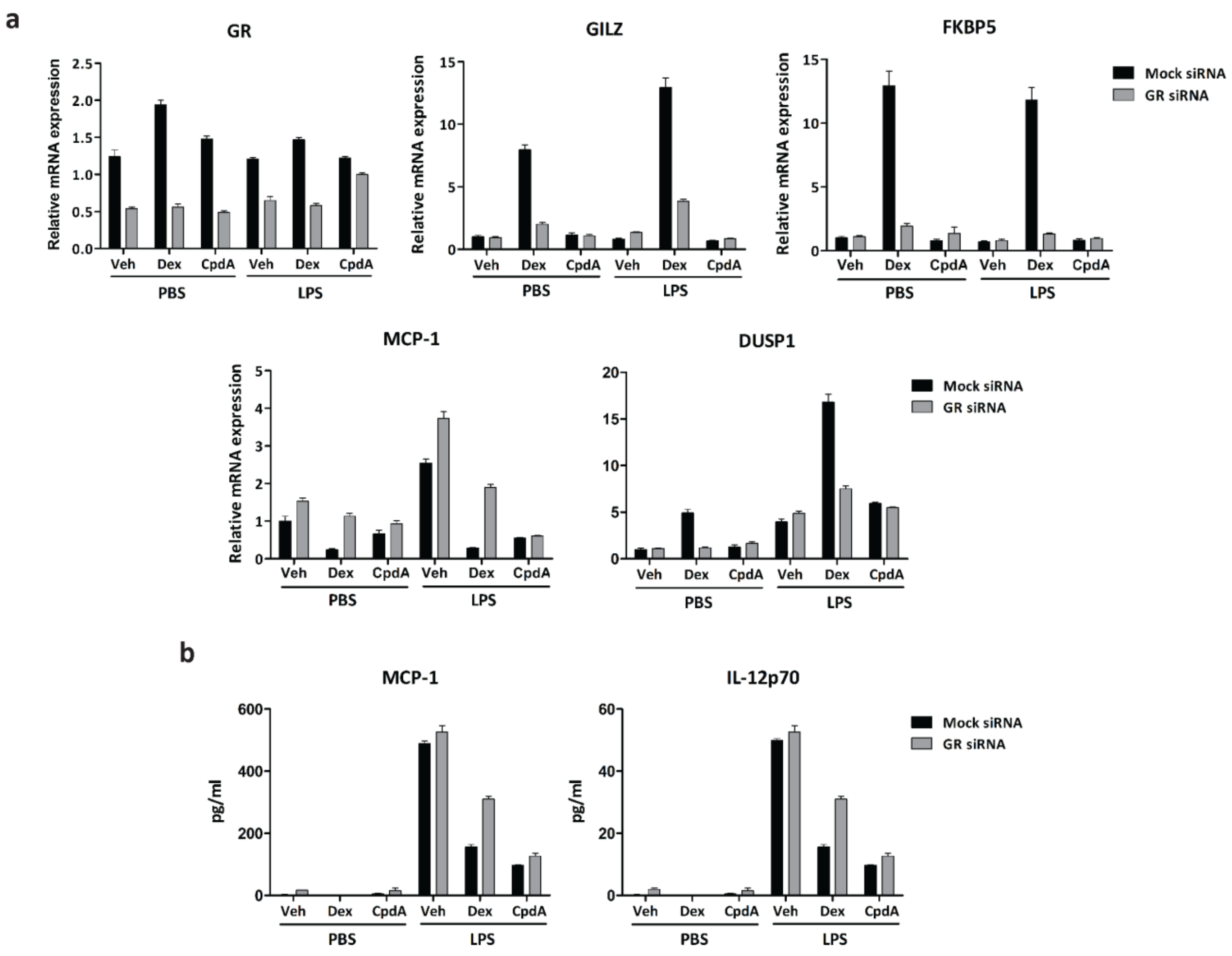

Supplementary Figure S4: The effects of CpdA on BMDC are not affected by GR knockdown. For efficient RNA interference, $3x10^5$ cells were transfected with 150 nM mouse GR siRNA (Dharmacon) or 150 nM non-silencing control scrambled (mock) siRNA (RL, Dharmacon) by using the Amaxa® Mouse Dendritic Cell Nucleofector® Kit (Lonza). Sixteen hours after transfection, cells were pre-treated with vehicle, CpdA 10µM or Dex 0.1µM for 1h before they were stimulated with LPS or PBS. **a)** RNA was isolated and levels of GR, GILZ, FKBP5, MCP-1 and DUSP1 mRNA were measured by qRT-PCR. **b)** supernatants were collected and secretion of MCP-1 and IL-12p70 was determined by ELISA. Data is presented as mean ± SD of different determinations from one representative (different than the presented in Fig. 6) out of three independent experiments.
